# A retrospective study on the efficacy of kyphoplasty with a curved-angle needle in addressing osteoporotic vertebral fractures at early versus delayed stages

**DOI:** 10.1097/MD.0000000000047535

**Published:** 2026-02-06

**Authors:** Chunlei Wu, Wen Hu, Hao Wang, Jun Liu

**Affiliations:** a Tianjin Medical University, Tianjin, P.R. China; b Department of Orthopedics, The 983rd Hospital of the Joint Logistic Support Force, Tianjin, P.R. China; c Department of Joint, Tianjin Hospital, Tianjin, P.R. China.

**Keywords:** delayed surgery, early surgery, osteoporotic vertebral compression fractures, percutaneous curved vertebroplasty

## Abstract

This study aims to assess the clinical efficacy of percutaneous curved vertebroplasty (PCVP) in the treatment of osteoporotic vertebral compression fractures (OVCFs) at 2 specified stages: early (symptom-to-surgery time ≤2 weeks) and delayed (symptom-to-surgery time >2 weeks). A retrospective analysis was conducted on 111 patients with OVCF who underwent PCVP at the 983rd Hospital of the Joint Logistic Support Force from June 2018 to June 2023. The patients were categorized into the early group (n = 60, symptom-to-surgery time ≤2 weeks) and delayed group (n = 51, symptom-to-surgery time >2 weeks) based on the interval from pain onset to surgical intervention. Demographic data were collected for both groups. The visual analog scale and Oswestry disability index were used to evaluate surgical efficacy. Perioperative complications were recorded. The kyphotic angle and vertebral height of the affected vertebra were measured preoperatively and postoperatively to assess the vertebral height recovery and kyphotic angle correction. A total of 217 patients were screened, of whom 106 were excluded (39 cases under the age of 60, 57 cases were unable to undergo surgical treatment due to systemic diseases, 10 cases with incomplete follow-up data), resulting in 111 eligible patients. Baseline characteristics were comparable between groups: early group (n = 60; age 73.03 ± 7.76 years; 12 males and 48 females) versus delayed group (n = 51; age 75.20 ± 6.63 years; 12 males and 39 females) (all *P* > .05). At the 12-month follow-up, the visual analog scale scores showed significant differences: early group (preoperative 8.12 ± 0.46 to postoperative 0.93 ± 0.52) versus delayed group (preoperative 6.55 ± 0.54 to postoperative 1.84 ± 0.37) (intergroup *P* < .001). The Oswestry disability index also demonstrated significant improvement: early group (preoperative 45.75 ± 1.58 to postoperative 11.68 ± 4.05) versus delayed group (preoperative 45.25 ± 1.98 to postoperative 15.59 ± 4.84) (intergroup *P* < .001). Regarding kyphotic angle correction, the early group showed a correction of −5.65° ± 1.93° compared to −0.76° ± 1.92° in the delayed group (*P* < .001). The anterior vertebral height recovery was greater in the early group (3.73 ± 1.71 mm) than in the delayed group (0.61 ± 1.57 mm) (*P* < .001). The complication rate was significantly lower in the early group at 10% (6/60) compared to 35.2% (18/51) in the delayed group (*P* = .001). Among elderly patients with OVCF aged >60 years and without contraindications, early PCVP performed within 2 weeks of symptom onset is associated with greater pain relief, enhanced functional improvement, restoration of vertebral height, and reduced complication rates compared to delayed surgery. Owing to the retrospective and observational nature of this study, causal inferences were constrained. Nevertheless, these findings support the consideration of early PCVP as a beneficial treatment strategy for eligible patients.

## 1. Introduction

Osteoporotic vertebral compression fractures (OVCFs) arise when the strength of the vertebrae diminishes, resulting in compression fractures from minimal trauma or in the absence of an obvious external force.^[1]^ This condition leads to a reduction in the vertebral height. If untreated, it may progress to kyphotic deformity over time, which can cause persistent thoracolumbar pain, increase the likelihood of refractures in the adjacent vertebrae,^[2,3]^ and significantly impair the quality of life of the elderly. Due to decreased bone mineral density (BMD) and heightened bone brittleness in the vertebral bodies, sparse trabecular bone is susceptible to fracture and may collapse under external forces.^[4,5]^ Many elderly individuals frequently experience postural lumbodorsal pain, which can obscure the diagnosis of insidious vertebral compression fractures, leading to insufficient attention and subsequent delays in diagnosis and treatment.^[6]^ Furthermore, once a vertebral fracture occurs, the absence of systematic treatment markedly elevates the risk of refractures in other vertebral bodies.^[7,8]^ OVCFs can result in severe kyphotic deformities of the spine, significantly compromising cardiopulmonary function^[9]^ and posing a substantial threat to patients. Therefore, a standardized treatment approach is of utmost importance. The clinical options for managing osteoporotic OVCFs include conservative and surgical intervention. Conservative treatment typically necessitates prolonged bed rest, which can lead to various complications, such as pressure ulcers, thrombosis, pneumonia, exacerbated calcium loss, and nonunion of fractures, known as Kummell disease.^[10]^ Moreover, many patients struggled to tolerate extended periods of bed rest. Vertebroplasty has emerged as the preferred treatment for elderly patients with OVCFs because of its minimal invasiveness and rapid recovery. This procedure effectively prevents the progression of kyphotic deformities by enhancing vertebral strength and restoring vertebral morphology while also providing significant pain relief.^[11]^

Currently, percutaneous vertebroplasty is the preferred minimally invasive treatment for OVCFs.^[12]^ Percutaneous curved vertebroplasty (PCVP), an advanced iteration of percutaneous vertebroplasty, offers several advantages, including the ability to access the contralateral side through unilateral puncture, multipoint injection of bone cement, and enhanced uniformity in cement distribution.^[13]^ Compared to traditional bilateral puncture, PCVP simplifies the procedure and reduces the need for intraoperative fluoroscopy.^[14]^ Additionally, it facilitates precise multipoint injection and optimizes bone cement distribution while minimizing the risk of leakage, thereby providing significant benefits for both physicians and patients.

However, consensus on the optimal surgical timing to achieve the best treatment outcomes remains elusive. The physiological basis for timing-dependent efficacy is not fully understood. Early surgery (≤2 weeks) takes advantage of the inflammatory phase of fracture healing, whereas delayed surgery (>2 weeks) may lead to complications such as fracture malunion, calcium deposition, and decreased vertebral compliance.^[15]^ In light of these uncertainties, this retrospective study compared the efficacy of early and delayed PCVP for OVCFs. This study aimed to fill the gap in PCVP-specific timing data and provide evidence-based guidance for clinical practice.

## 2. Methods

### 2.1. Study design and participants

A retrospective cohort study was conducted involving symptomatic patients with OVCF admitted to the 983rd Hospital of the Joint Logistic Support Force from June 2018 to June 2023.

*Inclusion criteria:* Age >60 years; OVCF diagnosed via X-ray (vertebral compression ≥20%) and magnetic resonance imaging (fat-suppressed T2-weighted sequences indicating bone marrow edema, confirming acute or subacute fracture); Osteoporosis confirmed by dual-energy X-ray absorptiometry (BMD T-score ≤ −2.5); Intact vertebral posterior wall (no radiological evidence of rupture); Absence of spinal cord or nerve root injury (normal neurological examination); and Completion of a 12-month clinical and radiological follow-up.

*Exclusion criteria:* Failure to meet the inclusion criteria; Severe systemic diseases (e.g., end-stage heart failure, coagulopathy, malignant tumors) that precluded surgery; Interrupted follow-up or incomplete clinical/radiological data; Prior spine surgery at the fracture level; and pathological fractures (e.g., metastatic lesions).

*Group stratification:* Patients were categorized into 2 groups based on a predefined threshold of 14 days for symptom-to-surgery interval.

- *Early group:* Surgery performed ≤2 weeks after symptom onset (corresponding to the inflammatory phase of fracture healing, during which vertebral reducibility is preserved^[8]^).
- *Delayed group:* Surgery performed >2 weeks after symptom onset (subacute/chronic phase, characterized by progressive fracture consolidation^[15]^).

This threshold was established based on 2 primary factors: The physiology of fracture healing, where the inflammatory phase lasts 1 to 2 weeks, followed by the formation of granulation tissue and calcium deposition;^[8]^ and Clinical guidelines for vertebral augmentation, which advocate for early intervention in acute OVCFs to enhance reduction outcomes. Group allocation was determined solely by the duration from symptom onset to surgery, without consideration of patient characteristics or surgeon preferences.

### 2.2. Surgical procedure

All surgeries were conducted by the same senior surgical team, with each member possessing at least 5 years of experience in PCVP to ensure standardization. The patients were positioned prone with abdominal suspension to mitigate spinal compression. Manual postural reduction was performed in the early group to restore vertebral height. The surgical area was disinfected and draped in a sterile manner. Under C-arm fluoroscopic guidance, the puncture point was identified at the superolateral margin of the pedicle projection (10 o’clock position for the left pedicles and 2 o’clock position for the right pedicles). A puncture technique involving 1% lidocaine local anesthesia was performed, followed by the insertion of a curved cannula injector. Anteroposterior fluoroscopy was used to position the tip at the inner edge of the contralateral pedicle, while lateral fluoroscopy was utilized to ensure the tip was located at the anterior one-third of the vertebral body. During the “stringing phase,” 3 to 5 mL of polymethylmethacrylate cement was injected because this viscosity was optimal for distribution. The injection was halted when the cement approached the posterior wall of the vertebra or exhibited mild leakage into the intervertebral space; if leakage occurred, the procedure was paused for 20 seconds before resuming cautiously. Postoperative management included bed rest for 6 to 8 hours, followed by ambulation with a thoracolumbar brace, which was worn for 3 months. All the patients received antiosteoporotic therapy consisting of oral bisphosphonates, vitamin D, and calcium.

### 2.3. Outcome measures

*Primary outcomes:* Pain relief, assessed using the visual analog scale (VAS: 0–10 scale, where 0 indicates no pain and 10 represents the worst pain),^[16]^ measured preoperatively, as well as at 1 day, 3 months, and 12 months postoperatively; Functional improvement, evaluated using the Oswestry disability index (ODI: 0–100 scale, where 0 signifies no disability and 100 denotes severe disability),^[17]^ measured at the same time points.

*Secondary outcomes:* Recovery of vertebral height, determined by measuring anterior, central, and posterior vertebral heights on lateral radiographs (Δ = postoperative height-preoperative height); Correction of the kyphotic angle, defined as the angle between the lines connecting the upper and lower endplates of the injured vertebra on lateral X-rays (Δ = postoperative angle-preoperative angle; negative values indicate correction); Perioperative complications, including but not limited to bone cement leakage, adjacent vertebral fractures, infections, and allergic reactions.

### 2.4. Statistical analysis

Data were statistically analyzed using SPSS software (version 18.0; IBM Corp., Armonk).

*Descriptive statistics:* Normally distributed measurement data are expressed as *x* ± *s*, while categorical data are presented as n (%).

*Intergroup comparisons:* Independent-sample *t* tests were used for continuous data and chi-square tests were used for categorical data.

*Intragroup comparisons:* Paired *t* tests were conducted to compare the preoperative and postoperative outcomes.

*Sample size justification:* A post hoc power analysis for the primary outcome, specifically the difference in VAS scores, was performed with an effect size of d = 0.8 (large effect^[18]^), α = 0.05, and a power of 80%. The required sample size was determined to be 98 patients; the current sample size of 111 patients surpassed this requirement, thereby confirming its adequacy.

*Confounder adjustment:* Multivariable linear regression analysis was conducted to adjust for potential confounders, including age, sex, and fracture segment (single vs multi-segment), regarding the primary outcome of the 12-month VAS improvement.

All statistical tests were 2-tailed, and *P* < .05 was deemed statistically significant.

## 3. Results

### 3.1. Patient enrollment and baseline characteristics

Baseline characteristics were comparable between the groups, with no significant differences observed in age, sex, fracture segment, preoperative VAS score, or preoperative ODI (all *P* > .05, Table 1). The early group exhibited a shorter interval from symptom onset to surgery (4.73 ± 1.69 days) than the delayed group (36.86 ± 39.55 days).

**Table 1:** Comparison of baseline characteristics between groups.

Variable	Early group (n = 60)	Delayed group (n = 51)	t/*χ*^2^	*P*
Age (years)	73.03 ± 7.76	75.20 ± 6.63	-1.563	0.121
Gender (male/female)	12 (20.0%)/48 (80.0%)	12 (23.5%)/39 (76.5%)	0.203	0.653
Fracture segment			0.011	0.918
Single-segment	43 (71.7%)	37 (72.5%)		
Multi-segment	17 (28.3%)	14 (27.5%)		
Preoperative VAS (points)	8.12 ± 0.46	6.55 ± 0.54	15.872	<0.001
Preoperative ODI (%)	45.75 ± 1.58	45.25 ± 1.98	1.551	0.125
Symptom-to-surgery interval (days)	4.73 ± 1.69	36.86 ± 39.55	-6.892	<0.001

ODI = Oswestry disability index, VAS = visual analog scale.

### 3.2. Surgical outcomes

*Functional outcomes (VAS and ODI):* Both groups demonstrated significant improvements in the VAS and ODI scores postoperatively (intragroup *P* < .001). However, the early group exhibited superior outcomes at all the follow-up time points (Tables 2 and 3). At the 12-month mark, the VAS score in the early group (0.93 ± 0.52) was nearly half that of the delayed group (1.84 ± 0.37), reflecting a mean reduction of 7.19 ± 0.68 points compared to 4.71 ± 0.61 points in the delayed group.

**Table 2:** VAS scores at different time points (*x* ± *s*, points).

Time point	Early group	Delayed group	Intergroup *P* value
Preoperative	8.12 ± 0.46	6.55 ± 0.54	<.001
Postoperative 1 day	2.20 ± 0.44	2.63 ± 0.60	<.001
Postoperative 3 months	1.52 ± 0.54	2.06 ± 0.24	<.001
Postoperative 12 months	0.93 ± 0.52	1.84 ± 0.37	<.001

VAS = visual analog scale.

**Table 3:** ODI scores at different time points (*x* ± *s*, %).

Time point	Early group		Delayed group	Intergroup *P* value
Preoperative	45.75 ± 1.58		45.25 ± 1.98	.125
Postoperative 1 day	17.63 ± 5.66		22.08 ± 5.82	<.001
Postoperative 3 months	13.93 ± 4.73		18.49 ± 5.27	<.001
Postoperative 12 months		11.68 ± 4.05	15.59 ± 4.84	<.001

ODI = Oswestry disability index.

*Radiological outcomes:* Vertebral height recovery and kyphotic angle correction were significantly greater in the early group (Table 4). This group demonstrated a mean anterior vertebral height recovery of 3.73 ± 1.71 mm, compared to 0.61 ± 1.57 mm in the delayed group (*P* < .001), resulting in a clinically meaningful difference of 3.12 mm that contributes to the restoration of spinal alignment and the reduction of kyphosis-related complications. Furthermore, kyphotic angle correction was more pronounced in the early group, with values of −5.65° ± 1.93° versus −0.76° ± 1.92° in the delayed group (*P* < .001). However, no significant difference in posterior vertebral height recovery was observed (*P* = .930).

**Table 4:** Changes in vertebral kyphotic angle and height (*x* ± *s*).

Outcome measure	Early group	Delayed group	*P* value
ΔKyphotic angle (°)	−5.65 ± 1.93	−0.76 ± 1.92	<.001
ΔAnterior vertebral height (mm)	3.73 ± 1.71	0.61 ± 1.57	<.001
ΔCentral vertebral height (mm)	3.68 ± 2.03	0.76 ± 1.91	<.001
ΔPosterior vertebral height (mm)	0.25 ± 1.41	0.12 ± 0.41	.930

*Complications:* The complication rate was significantly lower in the early group than in the delayed group (10% vs 35.2%, *P* = .001; Table 5). Primary complications included electrolyte disorders (early: 3 cases; delayed: 8 cases), anemia (early: 2 cases; delayed: 6 cases), and hypoproteinemia (early: 1 case; delayed: 4 cases), all of which were linked to prolonged bed rest in the delayed group. No severe complications such as spinal cord injury or fatal cement embolism were reported in either group.

**Table 5:** Surgical time and complications.

Variable	Early group (n = 60)	Delayed group (n = 51)	*t*/*χ*^2^ value	*P* value
Surgical time (min)	40.77 ± 9.88	45.59 ± 14.62	−1.251	.211
Complications (n, %)	6 (10.0%)	18 (35.2%)	10.408	.001

## 4. Discussion

**Figure 1.**
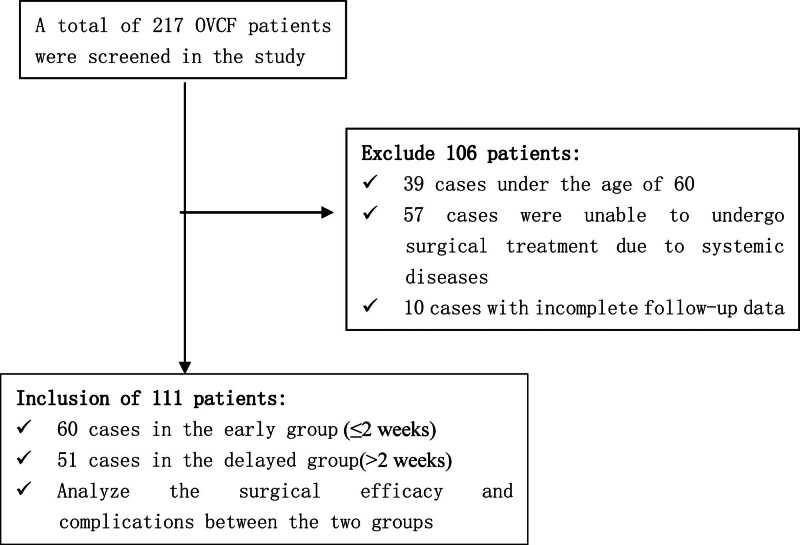
STROBE-compliant patient flow chart.

This retrospective study compared early and delayed PCVP for OVCFs in 111 elderly patients (age >60 years). These results indicate that early intervention (≤2 weeks after symptom onset) is associated with greater pain relief, improved functional outcomes, favorable radiological results, and reduced complication rates. These findings fill a crucial gap in the timing data specific to PCVP, and are consistent with the physiological principles underlying fracture healing (see Fig. 1).

### 4.1. Interpretation of key findings

*Pain relief and functional recovery:* The early group demonstrated a clinically significant reduction of 7.19 points in the VAS compared to a reduction of 4.71 points in the delayed group. This difference may be attributed to 2 mechanisms: The early stabilization of fracture micro-movements, which reduces nerve root irritation;^[18]^ and The heat generated during bone cement polymerization (50–60 °C), which destroys pain-sensitive nerve endings in the vertebral body, thereby preventing the development of chronic pain pathways.^[19]^ Preoperative VAS scores were higher in the early group (8.12 ± 0.46 vs 6.55 ± 0.54), indicating acute fracture pain, while patients in the delayed group experienced partial pain relief due to bed rest, consistent with prior observations.^[20]^ Multivariable regression analysis confirmed that early surgery was an independent predictor of pain relief, underscoring a direct association with timing rather than confounding factors.

*Radiological outcomes:* Early restoration of the anterior vertebral height to 3.73 ± 1.71 mm (compared to 0.61 ± 1.57 mm) is clinically significant. This improvement effectively mitigates the progression of kyphosis by enhancing vertebral height and restoring vertebral morphology, which can significantly alleviate pain^[11]^ and lower the risk of cardiopulmonary compromise. This advantage arises from the preservation of vertebral reducibility during the inflammatory phase; in contrast, delayed patients often experience fracture malunion and calcium deposition, which restrict reduction.^[21]^ Furthermore, improved spinal alignment minimizes altered loading on the adjacent vertebrae, thereby reducing the risk of refracture.^[22]^

*Complication rates:* The complication rate in the delayed group was 3-fold higher (35.2% vs 10%), associated with factors such as prolonged bed rest in elderly patients. Early surgery facilitates rapid mobilization (6–8 hours postoperatively), thereby mitigating these risks, which is a critical advantage for frail patients.

In this study, PCVP was employed to treat OVCFs. The results demonstrated that this surgical technique significantly alleviated pain and enhanced the patients’ daily activities in both the early and delayed treatment groups. Importantly, our findings underscore the significance of early interventions. This study contributes to the existing literature by affirming that timing is a critical variable, irrespective of the vertebroplasty technique utilized.

### 4.2. Limitations

This study has several limitations that warrant acknowledgments. *Retrospective, single-center design:* Selection bias may be present (e.g., healthier patients undergoing early surgery), despite multivariable adjustments for key confounders; *Absence of a control group:* We did not compare PCVP to conservative treatments (e.g., bed rest and bracing), which limits conclusions regarding the absolute efficacy of surgery versus nonsurgical management; *Subjective outcome measures:* VAS and ODI rely on patient self-reports, which may be affected by age-related cognitive decline or recall bias; *Limited covariate adjustment:* While we adjusted for age, sex, and fracture segment, other factors (e.g., comorbidity burden, BMD severity, and medication use) may have impacted the outcomes; *Lack of long-term refracture data:* We did not monitor refracture rates beyond 12 months, which is a crucial outcome in patients with OVCF; and *Potential unmeasured confounders:* Factors such as patient compliance with postural reduction or brace use were not formally evaluated. Future prospective randomized controlled trials with larger sample sizes, extended follow-up, and conservative treatment arms are necessary to validate these findings.

## 5. Conclusions

Among elderly patients with OVCF aged >60 years and without contraindications, early PCVP performed within 14 days of symptom onset is associated with greater pain relief, functional improvement, restoration of vertebral height, correction of the kyphotic angle, and reduced complication rates compared to delayed surgical intervention. Owing to the retrospective and observational nature of this study, causal inferences cannot be drawn; however, these findings suggest that early PCVP may be a favorable treatment strategy for eligible patients. Clinicians should prioritize prompt evaluation and intervention for elderly OVCF patients to optimize outcomes, while carefully weighing the surgical risks against the potential harm of delayed treatment. Future randomized controlled trials are necessary to validate these findings and stablish definitive guidelines regarding the timing of PCVP in OVCFs.

## Author contributions

**Resources:** Hao Wang.

**Supervision:** Jun Liu.

**Writing – original draft:** Wen Hu, Chunlei Wu.

**Writing – review & editing:** Wen Hu, Chunlei Wu.
